# Genetic Diversity Analysis and Comprehensive Evaluation of “M82” in EMS-Mutagenized Tomato

**DOI:** 10.3390/genes16020179

**Published:** 2025-02-01

**Authors:** Yanchao Yang, Zhanming Tan, Shuang Liang, Wei Cheng, Yihuan Sun, Yunxia Cheng, Yu Song, Yongming Wang, Jialong Wu, Qi Wang

**Affiliations:** 1 College of Horticulture and Forestry Sciences, Xinjiang Production & Construction Corps Key Laboratory of Facility Agriculture, National and Local Joint Engineering Laboratory of High Efficiency and High Quality Cultivation and Deep Processing Technology of Characteristic Fruit Trees in Southern Xinjiang, Tarim University, Alar 843300, China; 10757223042@stumail.taru.edu.cn (Y.Y.); 17362195960@163.com (S.L.); 18293233864@163.com (W.C.); 19039702334@163.com (Y.S.); chengyunxia2018@163.com (Y.C.); wjl955yyc@163.com (J.W.); 17699664091@163.com (Q.W.); 2 Institute of Crop Germplasm Resources, Xinjiang Academy of Agricultural Sciences, Urumqi 830000, China; songyu150@163.com; 3 Agricultural Science Research Institute of the First Division, Alar 843300, China; wym19802006@126.com

**Keywords:** processed tomato, EMS mutagenesis, genetic diversity

## Abstract

**Background:** Ethyl methyl sulfonate (EMS) mutagenesis is widely used because of its advantages of inducing point mutations and no need for genetic transformation. To identify germplasm resources of processed tomatoes with superior comprehensive traits suitable for cultivation in Xinjiang. Methods: In this study, tomato seeds were treated with 2% EMS reagent for 12 h, 21 quality traits and 20 quantitative traits of 33 processed tomatoes derived from EMS-mutagenized“M82”were evaluated. Results: The results indicated that for traits such as hypocotyl color, growth habit, plant type, leaf type, and leaf shape, the range of quantitative trait variation was 8.45–37.25%, with a genetic diversity index ranging from 1.25 to 2.07. Conclusions: Cluster analysis of quantitative traits categorized the 33 EMS-mutagenized “M82” processed tomato resources into five groups: Group I contained 22 robust germplasm samples; Group II consisted of a single potential high-quality germplasm; Group III comprised five germplasm with a small and extreme plant type; Group IV included four high-yield germplasm; and Group V represented one moderate, conventional germplasm. Raw data from 15 quantitative traits across the 33 accessions were standardized using the “extreme method” to extract six comprehensive factors. The top 10 germplasm resources based on the comprehensive score were 76, 137, 97, 102, 19, 104, 21, 108, 17, and 147. It provides some theoretical basis for realizing the high-yield and high-quality cultivation and variety breeding of processed tomatoes in Xinjiang.

## 1. Introduction

Processing tomato (*Solanum lycopersicum* L.) is a vital cash crop. China is one of the three principal global production areas for processing tomatoes [1], with Xinjiang being the most significant planting and processing region within the country. The planting area for processing tomatoes in Xinjiang constitutes 75% [2] of the total area in China. In 2019, Xinjiang’s processing tomato planting area reached approximately 33,400 hm^2^, producing 38,903,700 t, which accounted for 90% [3] of the region’s total output. The processing tomato variety “M82” is a model variety with a relatively clear genetic background. Currently, there is already an in-depth understanding of its basic growth characteristics, fruit traits, and physiological characteristics. In subsequent research, the ability to accurately identify new traits resulting from mutagenesis enables a more precise determination that these traits are the outcome of mutagenesis rather than being caused by other environmental factors or natural variations within the variety itself [4]. Despite its importance, the development of new processing tomato varieties in China faces challenges such as limited germplasm resources and a lack of foundational materials [5]. Chemical mutagenesis has been long-term applied by researchers in breeding work due to its characteristics such as simple operation, high mutation frequency, mutation specificity, and pleiotropy. Chemical mutagenesis has been long-term applied by researchers in breeding work due to its characteristics such as simple operation, high mutation frequency, mutation specificity, and pleiotropy. Ethyl Methyl Sulfone (EMS) mutagenesis is extensively employed due to its ability to induce high-density point mutations in DNA without causing chromosomal breakage or aberrations. EMS mutagenesis is cost-effective, straightforward to perform, and capable of inducing mutations in many plant materials simultaneously. Furthermore, it achieves effective mutagenesis with minimal technical complexity [6]. It plays an important role in research fields such as crop breeding, genetic improvement of ornamental plants, and the exploration of plant gene functions [7].

In tomatoes, Wang Yaling et al. [8] found that yellow-green leaf mutations induced by EMS mutagenesis were governed by recessive genes. For processing tomatoes, Ma Haixin et al. [9] utilized the “JW9” variety as a material and determined the optimal EMS treatment concentration and duration through gradient experiments (concentrations of 0%, 1%, 2%, 3%, 4% and durations of 6 h, 12 h). Their findings indicated that wet seeds absorbed EMS more efficiently. Yuehua Zhang et al. [10] observed that phenotypic variations induced by a 0.2% EMS solution were more diverse compared to those produced by 60Co-γ radiation mutagenesis. This demonstrates the advantages of EMS mutagenesis breeding, including high mutagenesis efficiency, diverse mutation frequency and types, strong stability, broad applicability, and ease of screening and identification. Although some mutation genes associated with key traits in processed tomatoes have been identified, the molecular regulatory mechanisms and their roles in metabolic pathways remain poorly understood. This gap limits the deeper understanding and effective utilization of mutant varieties. Current research predominantly focuses on the improvement of single traits, lacking systematic approaches to synergistically improve multiple important traits. This limitation makes it challenging to fulfill the demand for varieties with well-rounded traits required in processed tomato production. Mutants generated by EMS mutagenesis may have problems such as unstable mutations and complex inheritance, which pose difficulties for subsequent genetic analysis and variety breeding. It is necessary to conduct multi-generation observations and analyses of the mutants to determine the stability of their mutations and genetic patterns [11]. However, in recent years., insufficient evaluation of the genetic stability and long-term ecological adaptability of mutants across generations raises uncertainties about whether these mutated traits can reliably inherit and maintain superior performance under prolonged cultivation. The integration of modern breeding technologies, such as EMS mutagenesis, gene editing, and molecular marker-assisted selection, remains underdeveloped and underutilized in processed tomato breeding. This has hindered the achievement of more efficient and precise breeding goals by failing to capitalize on the strengths of these advanced techniques. This study aims to generate a batch of phenotypically variable mutants through EMS treatment of “M82” processed tomatoes, conduct a systematic analysis of their phenotypes, and identify favorable mutations in quantitative traits such as soluble solids content, soluble sugar content, and vitamin C content in their offspring. This effort seeks to enrich the germplasm resources available for processed tomato breeding. By assessing the quality traits and quantitative traits of “M82” processed tomatoes following EMS mutagenesis, the genetic differences and correlations among various traits will be examined. Additionally, the polymorphism information content of SNP sites will be calculated to explore genetic diversity patterns. A comprehensive evaluation system for the genetic diversity of “M82” processed tomatoes will then be established. This approach aims to select superior mutant materials with well-rounded traits, offering high-quality germplasm resources. The findings will provide a partial theoretical basis for achieving high-yield and high-quality cultivation and variety selection of processed tomatoes in Xinjiang. Subsequently, the parental materials with excellent traits can be crossed with other varieties to cultivate superior varieties with more comprehensive traits, thereby improving the yield and quality of tomatoes, increasing farmers’ income, and enhancing market competitiveness.

## 2. Materials and Methods

### 2.1. Test Site

(1) Tarim University Horticulture Experimental Station, First Division, Alar City (81°17′50″ E, 40°32′30″ N).
(2) Key Laboratory of Facility Agriculture in Southern Xinjiang.

### 2.2. Test Materials

(1) Non-mutagenized processing tomato “M82” and EMS-mutagenized M2 generation “M82” processing tomato.
(2) Fresh young leaves of mutant tomato plants processed by EMS mutation “M82”.

### 2.3. Test Design

#### 2.3.1. EMS Treatment of Xinjiang Processed Tomato Seeds

(1) EMS solution preparation:

To prepare the storage solutions A and B, 13.54 g of potassium dibasic phosphate and 22.822 g of dibasic phosphate were weighed and dissolved in distilled water to a final volume of 500 mL. These were labeled as solutions A and B, respectively. A mixture of 97.5 mL of solution A and 152.5 mL of solution B was prepared, adjusted to pH 7.0, and diluted to a final volume of 500 mL. A 2% EMS solution was prepared in a 0.1 mol/L phosphate buffer (pH = 7.0) at a *w*/*v* ratio.

(2) EMS solution treatment:

Full, mature tomato seeds were selected, soaked in clean water for 8 h, and then surface moisture was removed using filter paper. The seeds were treated with the prepared 2% EMS solution in an Erlenmeyer flask for 10 h on a shaker set to 200 rpm. The waste solution was neutralized with 5% sodium thiosulfate solution for 24 h to stop the mutagenesis and detoxify the solution. The seeds were then thoroughly rinsed with clean water and incubated at 28 °C to germinate, and the germination rate was recorded. Based on preliminary tests, a 2% EMS solution was used to treat tomato seeds for 12 h, resulting in a 50% lethality rate. The M1 generation seeds were harvested after treatment.

#### 2.3.2. Screening of Tomato Mutants

The M2 generation, as the largest segregating generation, exhibits the most pronounced phenotypic variation. In this study, both background materials and an M2 mutant library were planted at the Yuntai University Horticultural Experimental Station. In January 2023, 35 mutagenized lines were sown, with each line represented by 30 seeds planted in 32-cell trays containing a substrate mixture of peat:vermiculite:roseite in a 2:1:1 ratio. Seedlings were transplanted at the three-leaf, one-heart stage with a row spacing of 45 cm and a plant spacing of 35 cm. The experiment was designed as a randomized block design with three replicates. Each plot measured 20 m in length and 5 m in width, with 10 plants of each mutagenized line planted per plot alongside protective rows. Plant growth parameters, including plant height, stem thickness, internode spacing, and leaf count, were recorded during the seedling, growth, and maturity stages. Fruit appearance and intrinsic quality were also evaluated. Salt-tolerant tomato mutants were identified based on key agronomic traits.

#### 2.3.3. SNP Genetic Analysis of Mutant Plants

Illumina NovaSeqTM sequencing data (raw data) was processed and subjected to quality control, filtering out low-quality data to generate high-quality clean data. Using BWA-MEME v10.5software [12], clean data were aligned to the reference genome sequence, producing sequence location files (BAM files). The Best Practices workflow of GATK 4.5.0 software [13] was applied to correct the BAM files and tag SNPs. Variant function annotation was conducted using SnpEff 5.1d software [14] in conjunction with gene prediction information from the reference genome, yielding SNP functional annotations.

### 2.4. Test Method

At the fruit ripening stage of the second and third panicles, 10 representative plants were randomly selected from each replicate for observation. A total of 21 quality traits and 20 quantitative traits were recorded.

#### 2.4.1. Quality Traits

Quality traits were assessed using the direct observation method, referencing the “Specification and Data Standards for the Description of Tomato Germplasm Resources” [15] for statistics, grading, and assignment (Table 1).

#### 2.4.2. Quantitative Traits

(1) Longitudinal and transverse diameters of fruits: measured by vernier calipers;
(2) Single fruit weight: Weigh with electronic balance (1/1000 balance AE124);
(3) Vitamin C content: determined by molybdenum blue colorimetric method;
(4) Soluble solids content: determined by portable handheld refractometer [16];
(5) Soluble protein content: Coomassie brilliant blue G-250 staining method was used to determine [16];
(6) Nitrate content: determined by spectrophotometry [16];
(7) Total soluble sugar content: measured by anthrone colorimetric method [16];
(8) Organic acid content: determined by acid-base titration [16].

#### 2.4.3. SSR Molecular Labeling and Analysis

DNA from the samples was amplified using SSR-PCR with selected primers deemed suitable for analysis. Electrophoresis gel plates were dried, and band statistics were recorded. A binary 0/1 assignment method was used for band notation: the presence of a band was recorded as “1”, while its absence was recorded as “0”. A data matrix of 0 and 1 values was created for statistical analysis. Polymorphism analysis was carried out by processing the data from polymorphic bands identified through SSR labeling using the Hipstr 0.6.2 software.

### 2.5. Data Analysis

Data processing and statistical analyses were performed using Excel 2019 (Microsoft, 2019) and SPSS 22.0 (IBM, 2006) software. Quality traits were digitized, and the frequency and genetic diversity index for each quality trait were computed. Quantitative traits were evaluated by calculating their mean, maximum, minimum, range, standard deviation, and coefficient of variation. For the genetic diversity index, each trait was categorized into 10 grades: the first grade included values less than X − 2δ, with an intermediate interval of 0.5δ, and the 10th grade included values greater than X + 2δ, where X represented the mean of the quantitative trait, and δ was the standard deviation. The Shannon-Wiener index was employed to indicate the genetic diversity index, calculated using the formula *H*′ = −∑*Pi* lnPi, where *Pi* represents the probability of occurrence of a phenotypic trait indicator at the ith level.

## 3. Results

### 3.1. Frequency Distribution and Statistical Analysis of Phenotypes in M2 Generation Population of EMS-Mutagenized Tomato “M82”

A total of 33 plots and 1020 individual plants were viable in the M2 generation population of the EMS-mutagenized “M82”. Agronomic traits of these plants were observed, and statistical analyses yielded the following conclusions (Table 2). Mutations in the M2 generation population were observed across cotyledons, true leaves, inflorescences, stems, and fruits, spanning all stages of tomato growth. According to Table 2. the hypocotyl color of the phenotypes of different M2 generation populations was mainly green, accounting for 99.49%; The growth habit was mainly infinite, accounting for 70.77%. The plant type was mainly erect, accounting for 63.08%. The hairy stems and leaves were mainly long and thin, accounting for 59.49%. The leaf type was mainly potato leaf type, accounting for 49.47%. The shape of the leaves was mainly pinnate compound leaves, accounting for 71.79%. The leaves were mainly drooping, accounting for 51.79%. The leaf vein color was mainly green, accounting for 99.49%; The leaf fissures were mainly shallow, accounting for 79.49%. The inflorescence type was dominated by single inflorescence, accounting for 69.74%. The length of the columella was mainly shorter than that of the stamens, accounting for 70.77%. The pedicle was mainly delayered, accounting for 97.95%; The ridge structure of the fruit was mainly medium, accounting for 53.46%. The shape of the top of the fruit was mainly slightly concave, accounting for 32.41%. The shoulders of the fruits were mainly flat, accounting for 51.80%. The fruit shape was mainly 3 rounds, accounting for 43.49%. These findings suggest that the genetic background of the 34 mutagenic germplasm materials is relatively narrow.

### 3.2. Variation and Genetic Diversity Analysis of Quantitative Characters in M2 Population of EMS Mutated Tomato “M82”

Table 3 illustrates that all 15 quantitative traits exhibited varying degrees of variation. The highest coefficient of variation was observed in leaf area (37.25%), with a range of 143.75–754.08 cm^2^. The smallest variation was found in the fruit longitudinal diameter (8.45%), with a range of 28.60–45.88 mm. Traits with significant variation (coefficients above 20.00%) included protein content (36.33%), soluble sugar content (31.93%), leaf length (30.93%), organic acid content (28.82%), plant height (27.22%), yield (24.45%), yield per plant (24.40%), and internode length (23.13%). These eight traits exhibited substantial genetic variability, highlighting their potential value in breeding programs. The coefficients of variation for other traits ranged from 8.45% to 19.66%. The EMS-mutagenized processing tomatoes demonstrated extensive genetic variability in quantitative traits, providing a valuable germplasm foundation for developing new varieties and improving existing traits in processing tomatoes.

The genetic diversity index (H′) for the EMS-mutagenized population ranged from 1.25 to 2.07, with an average of 1.85, indicating a favorable overall genetic diversity (Table 3). The minimum soluble sugar content is 1.25; the maximum stem diameter is 2.07. The order of genetic diversity index from high to low is stem diameter (2.065) > VC content (2.018) > leaf length (2.017) > yield (1.968) > yield per plant (1.960) > longitudinal diameter of fruit (1.944) > leaf width (cm) (1.933) > Internode (mm) (1.931) > Organic acid content (1.9 23) > Fruit transverse diameter (1.900) > Protein content (1.822) > Plant height (cm) (1.731) > Leaf area (1.649) > Nitrate content (1.595) Soluble sugar content (1.249), it can be seen that EMS mutagenesis processed tomatoes show great differences in agronomic traits and contain rich genetic variation.

### 3.3. Detection and Annotation of Variation Loci in Tomato “M82” M2 Population Induced by EMS

To explore genetic diversity, the polymorphism information content of SNP loci was calculated. Sequencing sample data were used for population calling, and variant loci, including homozygous and heterozygous SNPs, were identified by comparing the samples with the reference genome. Conversion and transversion types of nucleotide substitutions were analyzed, and small fragment insertion and deletion (indel) events were counted to assess differences between the samples and the reference genome. A total of 46.07 million heterozygous SNP loci were identified across 32 samples (Table 4), with the conversion/transversion (Ts/Tv) ratio of SNP variants ranging from 1.20 to 1.21.

#### 3.3.1. SNP Detection

Single-Nucleotide Polymorphism (SNP) represents DNA sequence variations caused by single-nucleotide changes at the genome level. SNPs are among the most common genetic variations in the genome. SNP mutation types are classified as transitions and transversions: Transitions involve substitutions within the same base category (purine to purine or pyrimidine to pyrimidine). Transversions involve substitutions between different base categories (purine to pyrimidine or vice versa). Transitions are generally more frequent than transversions, resulting in a Ts/Tv ratio typically greater than 1, with specific values depending on the species studied.

The GATK Best Practices pipeline was employed to process alignment results (BAM files). SNP detection was carried out using the GATK Haplotyper method, applying filter conditions recommended by GATK parameters. SNP statistics were compared with the reference genome (M82 Genome Assembly and Annotation, SollycM82_v1, available at https://spuddb.uga.edu/SollycM82_v1_download.shtml 17 February 2023). The statistical results of SNPs in the samples are shown in Table 4.

#### 3.3.2. SNP Function Annotation

Table 5 illustrates that after removing duplicate loci across populations, a total of 46,070,107 SNP sites were identified among the 32 tomato samples. Annotation software was used to determine the positional information and functional impact of these SNP variation sites. The results are summarized in Table 5. Most SNPs were in intergenic regions, accounting for 42,600,902 sites (92.47%). SNPs within the exon regions represented 0.69% of the total, which included: Nonsynonymous mutations: 50,309 sites. Translation termination sites: 16,447 sites. Translation termination loss sites: 2540 sites. Synonymous mutations: 250,068 sites. Additionally, 7164 SNPs were located simultaneously in the 2 kb regions upstream and downstream of genes.

**Figure 1:**
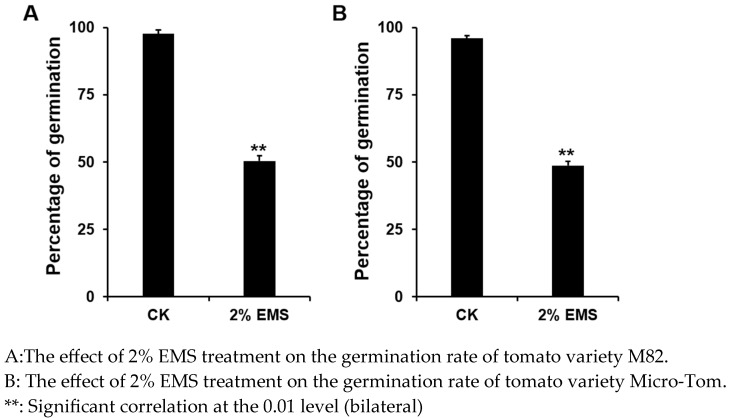
Effect of 2% EMS treatment on tomato germination rate.

**Figure 2:**
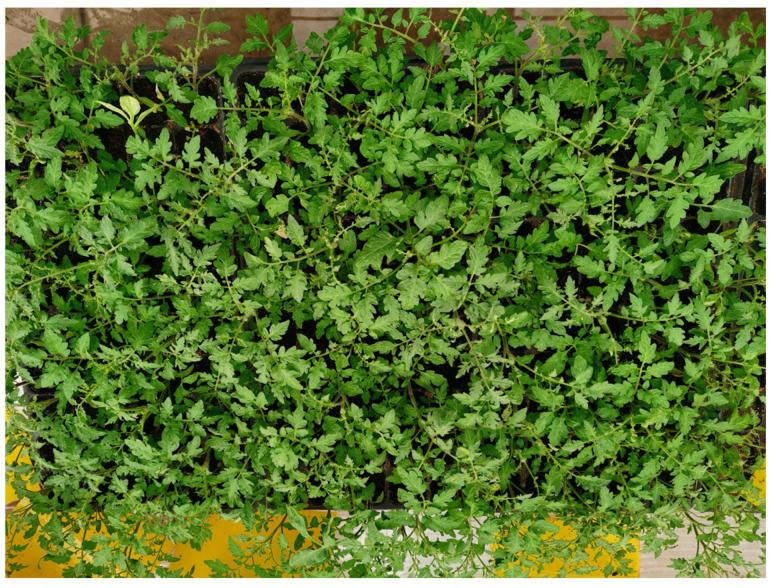
Effect of 2% EMS treatment on the phenotype of tomato “M82”.

**Figure 3:**
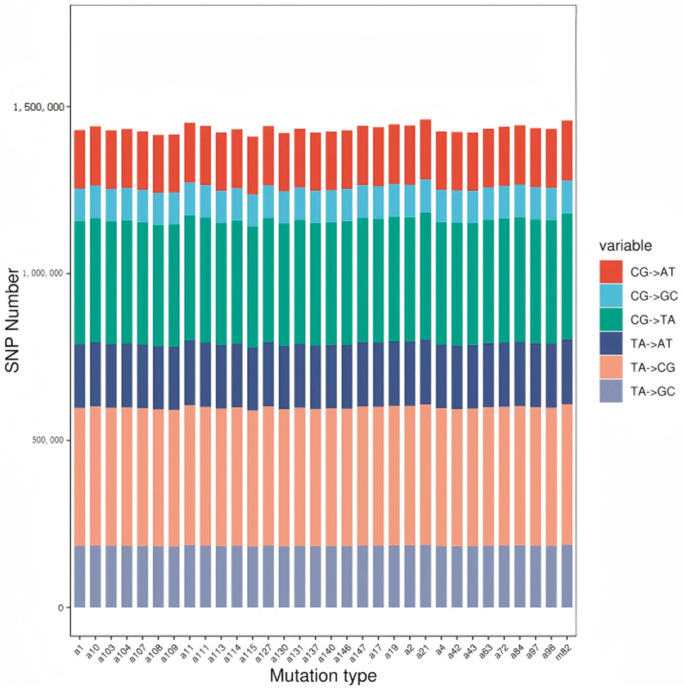
Statistics on SNP mutant base bias.

The base preference for different mutation types was determined by analyzing the nucleotide sequences before and after mutation at the SNP sites. Mutation types and preferences are shown in Figure 1, Figure 2 and Figure 3. The results indicate that SNP mutations predominantly involve A and T bases. The most common mutation types are T:A → C:G and C:G → T:A transitions.

### 3.4. Correlation Analysis of Quantitative Traits in Processed Tomatoes

**Figure 4:**
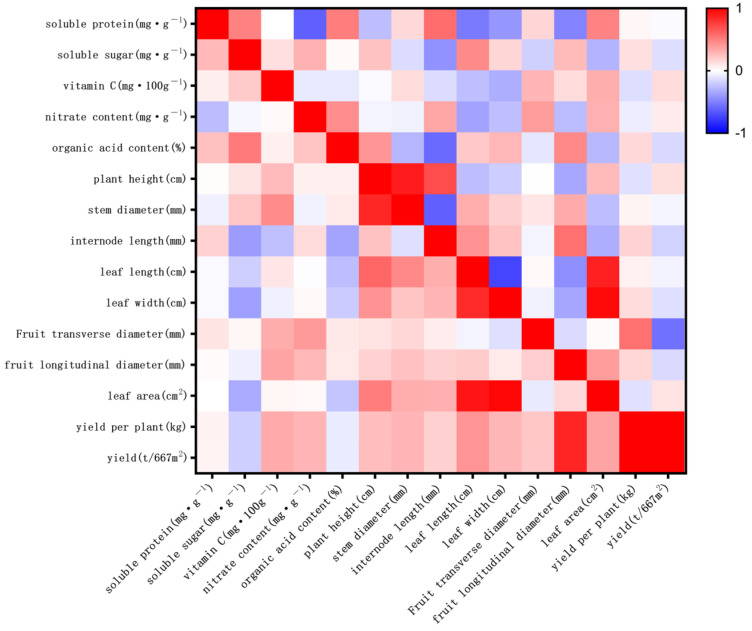
Quantitative trait correlation chart.

Figure 4 highlights the correlations among various traits of processed tomatoes subjected to EMS mutagenesis. Key findings include: Protein content: Exhibited weak correlations with other indices, indicating minimal influence of other traits on protein changes. Soluble sugar: Showed a significant positive correlation with organic acids (R^2^ = 0.514) and significant negative correlations with internode length (R^2^ = 0.3901) and leaf width (R^2^ = 0.3802). This suggests that higher soluble sugar levels are associated with higher organic acid content, shorter internodes, and narrower leaves. Vitamin C content: Positively correlated with stem diameter (R^2^ = 0.4533) and fruit longitudinal diameter (R^2^ = 0.3559), indicating that higher vitamin C content is linked to thicker stems and longer fruit. Nitrate content: Positively correlated with fruit transverse diameter (R^2^ = 0.3923), suggesting that higher nitrate content corresponds to wider fruit. Plant height: Strongly positively correlated with stem diameter (R^2^ = 0.8479), leaf length (R^2^ = 0.5998), and leaf area (R^2^ = 0.5062), indicating that taller plants tend to have thicker stems, longer leaves, and larger leaf areas. Internode length: Positively correlated with leaf length (R^2^ = 0.463), suggesting that longer internodes are associated with longer leaves. Leaf length: Showed strong positive correlations with leaf width (R^2^ = 0.8321) and leaf area (R^2^ = 0.9171) and significant positive correlations with yield (R^2^ = 0.4169) and yield per plant (R^2^ = 0.4186). This indicates that longer leaves with greater width and area contribute to higher yields. Leaf width: Strongly positively correlated with leaf area (R^2^ = 0.9762), showing that wider leaves contribute to larger leaf areas. Fruit longitudinal diameter: Positively correlated with yield (R^2^ = 0.8546) and yield per plant (R^2^ = 0.8538), suggesting that longer fruits contribute to higher yields. Leaf area: Positively correlated with yield per plant (R^2^ = 0.3544) and yield (R^2^ = 0.3561), indicating that larger leaf areas increase yields. Yield per plant: Perfectly correlated with yield (R^2^ = 1), confirming that higher yield per plant directly contributes to total yield.

### 3.5. Cluster Analysis

**Figure 5:**
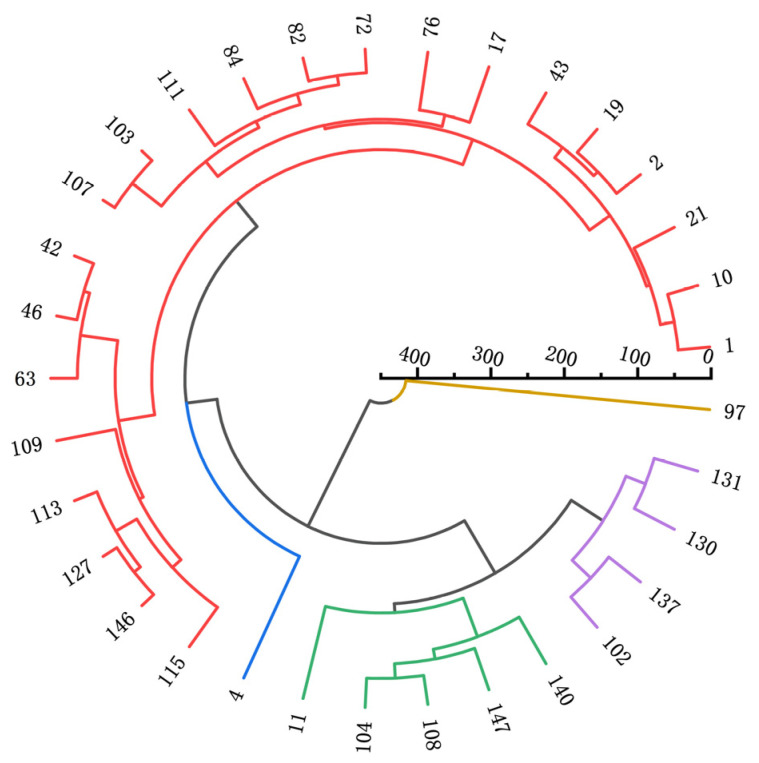
Statistical chart of clustering results.

As illustrated in Figure 5, the 33 EMS-induced processed tomato materials were classified into five major groups. Quantitative traits of each group were analyzed and clustered using Origin 2022 (Figure 1).

Group I: This group includes 22 materials and is characterized by higher protein content, plant height, stem diameter, internodes, leaf length, leaf width, and leaf area. These attributes define it as a robust plant germplasm. The phenotype of this group is relatively less influenced by environmental factors, making it a reliable resource for assessing genetic differences between strains or varieties. Robust germplasms are valuable for accurately reflecting genetic variations under varying conditions. Group II: Containing only one accession, this group is distinguished by lower fruit longitudinal diameter and yield but higher soluble sugar content, identifying it as a potentially high-quality germplasm. In processing tomato breeding, this germplasm can be crossed with high-yielding but less sweet varieties to develop new varieties with both high yield and improved sweetness, increasing overall fruit quality. Group III: This group includes five accessions characterized by lower plant height, stem diameter, internodes, leaf length, leaf width, and leaf area, representing extreme germplasm with smaller plant sizes. Small-sized germplasms are advantageous for dense planting, making them suitable for maximizing yield on limited land resources. Group IV: Comprising four accessions, this group is defined by higher yield and is categorized as high-yielding germplasm. These accessions are particularly beneficial for farmers aiming to increase agricultural output, thereby improving economic returns. Group V: This group contains one accession characterized by lower protein content, vitamin C content, and fruit transverse diameter. It represents conventional germplasm with moderate indicators. Such germplasms are typically adaptable to a wide range of environmental conditions, offering a stable genetic background for breeding programs.

### 3.6. PCA (Principal Component Analysis)

The raw data of 15 quantitative traits from 33 germplasm resources were standardized using the “range method” and subjected to PCA. The KMO test value of 0.700 and Bartlett’s sphericity test (*p* < 0.01) confirmed the suitability of the data for PCA. Six principal components (PCs) with eigenvalues ≥1 were extracted, explaining a cumulative variance of 85.95% (Table 6). First Principal Component (PC1): The eigenvalue for PC1 was 4.5959, contributing 30.64% to the total variance. Traits with high positive loadings included plant height (0.3041), stem diameter (0.2565), internode length (0.1746), leaf length (0.389), leaf width (0.3368), fruit longitudinal diameter (0.2834), leaf area (0.3735), yield per plant (0.3623), and yield (0.3628). These traits primarily reflected the influence of EMS mutagenesis on the growth characteristics of processed tomatoes, demonstrating a wide range of growth period adaptations in the ecological environment. Second Principal Component (PC2): PC2 had an eigenvalue of 2.69 and accounted for 17.98% of the variance. Key traits with high positive loadings included soluble protein (0.943), soluble sugar (0.32), vitamin C (0.35), nitrate content (0.198), and organic acid content (0.325). This component mainly represented quality factors influenced by EMS mutagenesis. Third Principal Component (PC3): With an eigenvalue of 2.08, PC3 contributed 13.87% of the variance. Traits such as leaf width, protein content, leaf area, vitamin C content, leaf length, organic acid content, soluble sugar, plant height, and stem diameter showed positive loadings. These traits indicated that increases in these characteristics promoted greater stem diameter. Fourth Principal Component (PC4): PC4, with an eigenvalue of 1.275 and a contribution of 8.52%, predominantly reflected protein composition factors. Fifth Principal Component (PC5): PC5 had an eigenvalue of 1.199 and contributed 8.00%, mainly representing nitrate content factors. Sixth Principal Component (PC6): The eigenvalue of PC6 was 1.040, contributing 6.94% to the variance. This component primarily reflected organic acid content factors.

### 3.7. Comprehensive Evaluation

Using the loading values and eigenvalues for each index, principal component coefficients were calculated to establish the linear relationships between principal components and the indices (Table 6). The equations for the principal components are as follows:

Y_1_ = −0.0085X_1_ − 0.1351X_2_ + 0.1438X_3_ + 0.0969X_4_ − 0.097X_5_ + 0.3041X_6_ + 0.2565X_7_ + 0.1746X_8_ + 0.389X_9_ + 0.3368X_10_ + 0.0777X_11_ + 0.2834X_12_ + 0.3735X_13_ + 0.3623X_14_ + 0.3628X_15_;

Y_2_ = 0.0943X_1_ +0.32X_2_ + 0.3502X_3_ + 0.1986X_4_ + 0.3252X_5_ + 0.0314X_6_ + 0.1733X_7_ − 0.2243X_8_ − 0.2134X_9_ − 0.3236X_10_ + 0.2952X_11_ + 0.3258X_12_ − 0.2843X_13_ + 0.2406X_14_ + 0.2393X_15_;

Y_3_ = 0.1174X_1_ + 0.3838X_2_ + 0.166X_3_ − 0.267X_4_ + 0.2156X_5_ + 0.397X_6_ + 0.4416X_7_ − 0.2474X_8_ + 0.1839X_9_ + 0.1066X_10_ − 0.1025X_11_ − 0.2676X_12_ + 0.1329X_13_ − 0.2601X_14_ − 0.2592X_15_;

Y_4_ = 0.8095X_1_ + 0.1635X_2_ − 0.1132X_3_ − 0.3223X_4_ + 0.1473X_5_ − 0.0823X_6_ − 0.203X_7_ + 0.3076X_8_ + 0.0132X_9_ + 0.0211X_10_ − 0.0853X_11_ + 0.1001X_12_ + 0.0323X_13_ + 0.0948X_14_ + 0.0939X_15_;

Y_5_ = 0.0906X_1_ + 0.0884X_2_ − 0.2844X_3_ + 0.5981X_4_ + 0.3574X_5_ + 0.181X_6_ − 0.0784X_7_ + 0.3199X_8_ + 0.0326X_9_ + 0.1177X_10_ + 0.4015X_11_ − 0.1687X_12_ + 0.0894X_13_ − 0.1776X_14_ − 0.1797X_15_;

Y_6_ = −0.138X_1_ + 0.16X_2_ − 0.3028X_3_ + 0.1765X_4_ + 0.4958X_5_ − 0.1781X_6_ − 0.1668X_7_ − 0.35 07X_8_ + 0.0682X_9_ + 0.2177X_10_ − 0.5219X_11_ + 0.1497X_12_ + 0.1553X_13_ + 0.1283X_14_ + 0.1295X_15_;

The comprehensive score (Z) was derived as the primary criterion for evaluating the effect of EMS mutagenesis on the 33 processed tomato materials. The comprehensive evaluation model for EMS mutagenesis effects on the phenotypic traits of “M82” was constructed as follows:

Z = 0.3063Y_1_ + 0.1798Y_2_ + 0.1387Y_3_ + 0.8522Y_4_ + 0.7998Y_5_ + 0.6937Y_6_.

The results (Table 7) showed that the comprehensive score (Z) for tomato germplasm resources ranged from −2.153 to 2.582. The three materials with the highest scores were 76, 137, and 97, with comprehensive scores of 2.582, 2.066, and 1.658, respectively.

## 4. Discussion

The genetic diversity of high-quality germplasm resources serves as the foundation for breeding programs and the development of new varieties. Analyzing genetic diversity and comprehensively evaluating germplasm resources are essential methods for identifying superior breeding materials and improving breeding efficiency. Previous studies have shown that the genetic diversity index (H’) ranges from 0.060 to 1.520, with most values falling between 0 and 1 for general traits. For quantitative traits, H’ values range from 0.680 to 2.070, with most values between 1 and 2. The coefficient of variation (CV) typically ranges from 3.53% to 96.56%, with most values below 40% [17]. In this study, EMS mutagenesis proved to be an effective genetic improvement method, inducing significant genetic variation in “M82” tomato. Genetic diversity analysis demonstrated that EMS mutagenesis enriched the genetic resources of processed tomatoes. This mutation method may cause gene mutations, chromosomal structural variations, and other genetic alterations, resulting in mutants with diverse phenotypes and genotypes. These findings align with the results reported by Zhang Long et al. [18].

Since EMS mutagenesis is non-directional, plants treated with EMS exhibit a wide range of mutation types, which manifest at various stages of growth and affect almost all tissues and organs [19]. In the M2 generation population of “M82” tomatoes subjected to EMS mutagenesis, mutations were observed across all growth stages. Key mutations included: Hypocotyl color: Primarily green. Growth habit: Mainly indeterminate. Plant type: Predominantly upright. Stems and leaves: Mostly long and sparse hairs. Leaf traits: Predominantly potato leaf type, pinnate compound leaf shape, drooping growth state, and green vein color. Leaf morphology: Primarily shallow clefts. Inflorescence: Mainly single inflorescence type. Style length: Generally shorter than stamens. Pedicel abscission layer: A significant mutation was observed. Similar studies have reported phenotypic variations in other horticultural crops such as mung bean [20], pumpkin [21], and watermelon [22]. These results highlight that EMS mutagenesis can generate diverse phenotypic variations in plant populations. The findings of this study contribute to the enrichment of crop germplasm derived from EMS mutagenesis, providing valuable resources for breeding and genetic improvement efforts.

Mutation types observed in materials obtained through EMS mutagenesis are diverse, with variations appearing across a wide range of plant traits [23,24]. Quan Hong Tran [25] et al. used EMS to induce inbred maize ML10, cloned a lock mutant after rapid sequencing and mapping, and identified a promoter deletion in ZmCLE7 (CLE 7)Similarly, Sun Mingyang et al. [26] performed EMS mutagenesis on American seed pumpkin and recorded 45 distinct phenotypic variations in the progeny, covering all growth stages and plant organs, with a total mutation frequency of 25.17%. In this study, variations were observed in 15 quantitative traits of EMS-mutagenized tomatoes, with a genetic diversity index (H’) ranging from 1.25 to 2.07, averaging 1.85. These results indicate a high level of genetic diversity overall. Thus, EMS mutagenesis provides a robust foundation for generating excellent germplasm resources to support the breeding of new varieties and the improvement of traits in processed tomatoes.

The correlation analysis of quantitative traits in EMS-mutagenized progeny revealed significant interrelations among various indices. Key findings include: Soluble sugar content: Significantly positively correlated with organic acid content and negatively correlated with internode length and leaf width. Vitamin C content: Significantly positively correlated with stem diameter and fruit longitudinal diameter. Nitrate content: Significantly positively correlated with fruit transverse diameter. Plant height: Significantly positively correlated with stem diameter, leaf length, and leaf area. Internode length: Significantly positively correlated with leaf length. Leaf length: Significantly positively correlated with both leaf width and leaf area. These results align with findings by U and Han Yunzhe [27] in rice, highlighting the significant correlations among quantitative traits in EMS-mutagenized progeny. Such correlations suggest that, in future breeding programs for processed tomatoes, targeted improvements can be made to related traits. For example, increasing soluble sugar and organic acid contents could lead to germplasm resources with improved flavor. Through strategic breeding efforts, germplasm with superior performance can be developed, meeting diverse agricultural and consumer demands.

Chemical induction methods in plant mutation breeding offer certain advantages over physical induction, including economic feasibility, high specificity, increased frequency of induction, and the tendency to produce point mutations [28]. EMS induction is particularly advantageous due to its high mutation rate, the ability to generate numerous point mutations, and its capacity to produce new genes and traits that are challenging to obtain through conventional breeding methods [29]. In this study, a comprehensive evaluation of the quality and quantitative traits of 33 EMS-induced processed tomato offspring derived from “M82” was conducted. Cluster analysis identified several superior or promising mutant offspring with desirable traits. Five distinct clusters were observed within the processed tomato offspring population. Cluster 1: Representing robust plant germplasm, this group displayed significantly higher protein content, plant height, stem thickness, internode length, leaf length, leaf width, and leaf area compared to other clusters. These findings align with results from studies by Zhao Miao [30] on EMS-induced amaranth and Zhang Ping [31] on EMS-induced tomato, indicating that EMS mutagenesis generally causes notable alterations in plant phenotypes. Cluster 2: This cluster comprised a single germplasm with lower fruit longitudinal diameter and yield but higher soluble sugar content. While there is limited research on EMS-induced offspring with these specific traits, this cluster provides new directions for breeding EMS-induced germplasm with desirable flavor characteristics. Cluster 3: Characterized by lower plant height, stem thickness, internode length, leaf length, leaf width, and leaf area, this cluster demonstrated phenotypic variations opposite to those in Cluster 1. These results are consistent with Liu Jialing’s [32] findings in EMS-induced Chinese cabbage. Cluster 4: Exhibiting high yield, this cluster aligns with Wang Junjie’s [33] study on EMS-induced millet, demonstrating that EMS induction can successfully produce high-yielding germplasm. Cluster 5: This cluster was characterized by lower protein content, vitamin C content, and fruit cross-sectional diameter, with moderate performance in other traits. Since no relevant studies have been reported on such trait combinations, this cluster provides a novel reference for future EMS-induced mutation breeding efforts.

The findings of this study demonstrate that processed tomato offspring subjected to EMS induction can be effectively categorized into distinct clusters through cluster analysis.

Primary data from 15 quantitative traits of 33 germplasm resources were standardized using the “range method” and analyzed via principal component analysis (PCA). Six principal components were extracted: PC1 primarily reflected the influence of EMS mutation on the growth period of processed tomatoes. PC2 and PC3 represented quality factors. PC4 was associated with protein component factors. PC5 captured nitrate component factors. PC6 reflected organic acid component factors. To date, no prior studies have reported principal component analysis of quantitative traits in EMS-mutagenized progeny, making these results a novel contribution to this field. They provide valuable references for future research directions in the study of EMS-mutant offspring.

Using the loadings and eigenvalues of each trait, principal component coefficients were calculated, establishing a linear relationship between principal components and each trait. This enabled the construction of a comprehensive evaluation model, expressed as: Z = 0.3063Y1 + 0.1798Y2 + 0.1387Y3 + 0.8522Y4 + 0.7998Y5 + 0.6937Y6. The comprehensive scores (Z) of the tomato germplasm resources ranged from −2.153 to 2.582. The three materials with the highest scores were 76, 137, and 97, with respective scores of 2.582, 2.066, and 1.658. This study not only identified germplasm resources with significant developmental potential but also provided valuable research materials and references for the comprehensive evaluation of EMS-mutagenized progeny.

The increase in genetic diversity provides a broader selection of options for breeding processing tomato varieties with superior traits. Different mutants exhibit variations in growth characteristics, fruit quality, and stress resistance. In this experiment, five distinct germplasm types were identified: robust plant vigor, potential high-quality germplasm, small extreme plant morphology, high-yield germplasm, and conventional germplasm. These groups form a foundation for further variety improvement and targeted breeding programs. Although mutagenesis increases genetic diversity, it may also result in undesirable mutations. Therefore, strict screening and evaluation are essential to identify mutant strains with potential agronomic value. High soluble sugar content is a crucial indicator of fruit quality, particularly in the food processing industry, where such fruits are highly valued despite their lower fruit length and yield. By hybridizing high soluble sugar content mutants with high-yielding and larger-fruit-length varieties, breeders can integrate desirable traits to develop superior cultivars.

## 5. Conclusions

The raw data from 15 quantitative traits across 33 germplasm resources were standardized using the “range method” and analyzed through principal component analysis. The top 10 germplasm resources based on comprehensive scores were 76, 137, 97, 102, 19, 104, 21, 108, 17, and 147. Cluster analysis of quantitative traits categorized the 36 EMS-mutagenized “M82” processed tomato resources into five groups: Group I: Robust plant germplasm (22 accessions). Group II: Potential high-quality germplasm (1 accession). Group III: Small plant-type extreme germplasm (5 accessions). Group IV: High-yielding germplasm (4 accessions). Group V: Moderate conventional germplasm (1 accession). This is of great significance in aspects such as variety screening and evaluation, genetic breeding, and agricultural production for guiding the breeding of processing tomato varieties, the selection of parents, the study of genetic laws of traits, the optimization of planting layout, and meeting different processing demands.

## Figures and Tables

**Table 1:** Description norms and data standards for processed tomato germplasm resources.

Character	Levels of Trait Description
Hypocotyl color	Hypocotyl color
habit of growth	1 = infinite growth and 2 = finite growth
plant type	1 = vine type 2= half-vine type 3 = upright
Stem leaf furry hair	0 = no 1 = short thin 2 = short dense 3 = long thin 4 = long dense
Leaf type	1 = common leaf type 2 = potato leaf type 3 = compound broad leaf type 4 = complex fine leaf type
Leaf shape	1 = pinnate complex leaves 2 = second rus pinnate complex leaves
The leaves are in a state of birth	1 = upright 2 = horizontal 3 = drooping
leaf color	1 = yellow-green 2 = light green 3 = green 4 = dark green
Leaf vein color	1 = colorless and 2 = green
Leaf crack engraved	0 = no 1 = shallow 2 = medium 3 = deep
inflorescence types	1 = single flowers 2 = single inflorescence 3 = double flowers 4 = multiple inflorescences
Clusters of flowers	0 = no 1 = Yes
The length of the column	1 = shorter than stamen 2 = nearly equal length to stamen 3 = longer than stamen
The color of the flower	1 = light yellow 2 = yellow 3 = orange yellow
The pedicle from the layer	0 = no 1 = Yes
Fruit surface edge ditch	0 = no 1 = light 2 = medium 3 = heavy
Fruit top shape	1 = deep concave 2 = micro concave 3 = round flat 4 = micro convex 5 = convex tip
Fruit shoulder shape	0 = no 1= flat 2 = microconcave 3 = deep concave
Fruit shape	1 = flat 2 = oblate 3 = circle 4 = high circle 5 = long circle 6 = oval 7 = peach circle 8 = pear-shaped 9 = long pear-shaped

**Table 2:** Mutation types and proportions of M2 generation tomatoes.

Quota	Evaluate										H’
	0	1	2	3	4	5	6	7	8	9	
Hypocotyl color		99.49%	0.51%								0.03
growth habit;		70.77%	29.23%								0.60
plant type		3.59%	33.33%	63.08%							0.78
Stem leaf furry hair		7.18%	16.41%	59.49%	16.92%						1.10
Leaf type		23.08%	49.74%	17.95%	9.23%						1.21
Leaf shape		71.79%	28.21%								0.59
The leaves are in a state of birth		21.03%	27.18%	51.79%							1.02
Leaf vein color		0.51%	99.49%								0.03
Leaf crack engraved		79.49%	19.49%	1.03%							0.55
inflorescence types		3.08%	69.74%	24.62%	2.56%						0.80
The length of the column		70.77%	4.10%	5.13%							0.53
The pedicle from the layer	2.05%	97.95%									0.10
Prism		18.01%	53.46%	17.17%	11.36%						1.19
blossom end		17.17%	32.41%	26.87%	13.30%	9.97%	0.28%				1.54
Fruit shoulder		0.28%	51.80%	41.00%	6.93%						0.91
Fruit shape	6.37%	6.37%	7.20%	43.49%	25.48%	1.39%	9.42%	0.28%			1.55

**Table 3:** Genetic diversity analysis of quantitative traits in tomato of M2 generation.

Shape and Properties	Average Value	Standard Deviation	Minimum Value	Maximal Value	Coefficient of Variation/%	H’
Protein (mg·g^−1^)	2.21	0.68	0.57	3.41	30.93%	2.02
Soluble sugar (mg·g^−1^)	92.98	26.80	36.24	147.98	28.82%	1.92
vc(mg·100·g^−1^)	31.42	6.18	17.56	49.38	19.66%	1.60
Nitrate (mg·g^−1^)	0.31	0.06	0.21	0.42	18.38%	2.02
organic acid (%)	0.01	0.00	0.01	0.01	18.02%	1.93
plant height (cm)	37.89	10.31	23.88	76.00	27.22%	1.71
Stem coarse (mm)	10.91	3.48	7.41	28.36	31.93%	1.25
internode (mm)	27.39	9.95	11.65	63.06	36.33%	1.82
Leaf length (cm)	21.00	3.26	14.94	27.80	15.51%	2.07
Leaf width (cm)	23.66	5.47	13.74	38.75	23.13%	1.93
Fruit cross diameter (mm)	38.88	3.32	29.18	45.78	8.55%	1.65
Fruit longitudinal diameter (mm)	38.94	3.29	28.60	45.88	8.45%	1.90
Leaf area (cm^2^)	357.96	133.33	143.75	754.08	37.25%	1.94
yield per plant (kg)	0.90	0.22	0.34	1.40	24.40%	1.96
yield (t/667 m^2^)	4.389	1.073	1.667	6.804	24.45%	1.97

**Table 4:** SNP statistics.

Sample ID	SNP Number	Transition	Transversion	Ts/Tv	Heterozygosity Number	Homozygosity Number
a1	34,179	21,069	13,111	1.61	8660	119,407
a10	32,473	19,361	13,115	1.48	8203	120,260
a103	32,130	19,130	13,004	1.47	8190	119,811
a104	30,799	18,316	12,487	1.47	7569	120,600
a107	31,364	18,624	12,743	1.46	7810	120,189
a108	31,144	18,501	12,644	1.46	7314	120,623
a109	37,582	23,954	13,628	1.76	10,039	117,627
a11	31,920	18,957	12,966	1.46	8501	120,158
a111	38,000	24,125	13,878	1.74	10,877	117,446
a113	31,285	18,546	12,739	1.46	7968	120,293
a114	31,302	18,714	12,590	1.49	7625	120,634
a115	34,321	21,315	13,008	1.64	7972	119,332
a127	34,264	20,937	13,328	1.57	9381	118,944
a130	36,470	22,437	14,035	1.60	9414	118,639
a131	35,320	22,109	13,214	1.67	8676	119,527
a137	33,729	20,814	12,917	1.61	7776	120,175
a140	31,602	18,718	12,885	1.45	7662	120,094
a146	39,720	24,614	15,108	1.63	11,963	115,902
a147	33,145	19,988	13,159	1.52	8643	119,604
a17	31,346	18,593	12,756	1.46	8399	119,834
a19	32,493	19,378	13,116	1.48	7732	120,468
a2	31,746	18,841	12,907	1.46	8999	119,381
a21	39,247	25,254	13,994	1.80	11,357	117,239
a4	31,205	18,576	12,633	1.47	8458	119,604
a42	37,534	23,828	13,708	1.74	10,546	117,464
a43	30,579	18,244	12,336	1.48	8229	119,869
a63	31,526	18,763	12,763	1.47	8065	120,127
a72	33,738	20,454	13,286	1.54	8948	119,420
a84	32,573	19,637	12,940	1.52	9789	118,435
a97	32,103	19,277	12,829	1.50	8039	120,136
a98	34,814	20,927	13,889	1.51	8538	119,693
m82	31,113	18,536	12,578	1.47	8436	120,126

Sample ID: Sample number; SNP Number: The number of detected SNPs indicates the nucleotide variation between the material and the reference genome; Transition: the number of SNPs converted; Transition: the number of SNPs transverted; Ts/Tv: the ratio of Transition SNPs to Tranversion SNPs; Heterozygosity Number: the total number of SNP sites heterozygous; Homozygosity Number: the total number of SNP sites homozygous.

**Table 5:** Distribution information statistics table of population SNP.

Classify;		Number
Upstream		119,731

exon	Stopgain; Stoploss; synonymous; nonsynonymous	16,447
		2540
		250,068
		50,309
Intronic		2,490,078

Splicing		63,113

Intergenic		42,600,902

UTR5		16,020
UTR3		201,073
UTR5;UTR3		708
RNAncRNA		0

Downstream		2,259,742

upstream/downstream		7164


**Table 6:** Eigenvalues and eigenvectors of the first six principal components.

Principal Components	PC1	PC2	PC3	PC4	PC5	PC6
soluble protein (mg·g^−1)^	−0.0085	0.0943	0.1174	0.8095	0.0906	−0.138
soluble sugar (mg·g^−1)^	−0.1351	0.32	0.3838	0.1635	0.0884	0.16
vitamin C (mg·100 g^−1^)	0.1438	0.3502	0.166	−0.1132	−0.2844	−0.3028
nitrate content (mg·g^−1)^	0.0969	0.1986	−0.267	−0.3223	0.5981	0.1765
organic acid content (%)	−0.097	0.3252	0.2156	0.1473	0.3574	0.4958
plant height (cm)	0.3041	0.0314	0.397	−0.0832	0.181	−0.1781
stem diameter (mm)	0.2565	0.1733	0.4416	−0.203	−0.0784	−0.1668
internode length (mm)	0.1746	−0.2243	−0.2474	0.3076	0.3199	−0.3507
leaf length (cm)	0.389	−0.2134	0.1839	0.0132	0.0326	0.0682
leaf width (cm)	0.3368	−0.3236	0.1066	0.0211	0.1177	0.2177
Fruit transverse diameter (mm)	0.0777	0.2952	−0.1025	−0.0853	0.4015	−0.5219
fruit longitudinal diameter (mm)	0.2834	0.3258	−0.2676	0.1001	−0.1687	0.1497
leaf area(cm^2^)	0.3735	−0.2843	0.1329	0.0323	0.0894	0.1553
yield per plant (kg)	0.3623	0.2406	−0.2601	0.0948	−0.1776	0.1283
yield (t/667 m^2^)	0.3628	0.2393	−0.2592	0.0939	−0.1797	0.1295
eigenvalue	4.5959	2.6975	2.08	1.2784	1.1997	1.0405
Percentage%	30.6396	17.9835	13.8667	8.5227	7.9979	6.9367
cumulative variance%	30.6396	48.6232	62.4899	71.0126	79.0106	85.9473

**Table 7:** Comprehensive Evaluation of 33 EMS Mutagenic Resources.

No	Y(i,1)	Y(i,2)	Y(i,3)	Y(i,4)	Y(i,5)	Y(i,6)	Comprehensive Score	Ranking
1	−4.1276	−3.1328	0.9697	−0.946	−0.2613	−0.7985	−2.153080588	33
2	−0.5189	−0.1488	−0.1237	0.0585	0.5098	−0.8406	−0.250679506	21
4	−3.5646	2.4312	0.1686	−0.5215	0.2229	0.2375	−0.746654022	29
10	−0.6913	1.536	−0.7669	−1.0034	0.2275	0.8296	−0.0601571	17
11	0.192	−1.296	1.0441	0.0232	0.4714	0.8205	0.078116564	13
17	−1.3992	0.1854	2.4221	1.0352	1.8863	1.4457	0.325632936	9
19	1.4227	1.9766	−1.9854	−0.8859	0.4334	0.4097	0.58598926	5
21	1.6679	0.0883	−1.6885	2.2524	0.6845	−2.3038	0.441760608	7
42	−1.3106	2.7436	−0.7717	0.2159	0.4492	1.2593	0.147188246	12
43	−1.751	−2.1808	0.4705	−0.9792	−0.2801	0.0446	−1.124181177	32
46	−1.1475	1.835	−0.5937	1.6082	−1.9671	−0.9829	−0.223816498	18
63	−1.3001	1.1611	−1.1036	−2.3667	0.5849	0.105	−0.570367436	28
72	−0.4578	0.0246	0.1098	0.4105	−0.6461	−1.1796	−0.254961555	23
76	3.8114	4.0838	4.7951	−1.972	−1.3328	−1.0524	2.582355156	1
84	−0.6938	−0.8998	1.2316	−0.7712	−0.1704	−0.9343	−0.404638508	24
97	4.7153	−2.2508	1.5808	0.5188	1.9576	−0.5056	1.657867015	3
102	3.0236	−1.0974	0.2238	1.3934	−1.7504	−1.0101	0.77814445	4
103	1.1924	−0.9202	−2.2686	−0.6524	1.679	0.345	−0.014082201	14
104	0.7744	0.3587	0.4491	0.9438	−0.0807	0.8979	0.582127412	6
107	0.303	−0.7355	−0.3155	0.4699	0.5861	−0.4002	−0.02794376	15
108	0.112	−0.0916	0.9201	0.5422	1.0094	0.8043	0.381819711	8
109	−2.4298	0.5402	−0.988	0.4839	−1.0478	−1.1611	−1.055809554	30
111	−1.016	−0.3405	0.4858	0.7376	0.2658	0.287	−0.234024271	19
113	−0.6333	1.5486	−1.8312	0.0201	−0.8155	0.2305	−0.252475452	22
115	−2.4588	−1.5251	0.9184	0.4725	−0.6702	−0.7038	−1.119795192	31
127	−2.2782	0.7928	−0.0469	0.6239	0.7429	0.5647	−0.477269899	26
130	3.2974	−0.9876	−3.1388	−1.7432	0.033	−0.5627	0.247239802	11
131	1.8687	−2.4816	−0.2027	−2.8707	−0.975	0.1544	−0.248704195	20
137	4.4798	1.7242	−0.657	1.1328	0.2921	0.9229	2.065784387	2
140	1.279	−2.3221	−0.3305	0.9546	−3.3476	3.2751	−0.035766054	16
146	−1.4883	0.8114	−0.3427	0.0169	−0.4856	−0.3959	−0.491547832	27
147	0.1231	−0.5427	1.2617	0.329	0.9207	0.6783	0.306938125	10
M82	−0.9959	−0.8883	0.104	0.4688	0.8738	−0.4805	−0.435099898	25

## Data Availability

The data presented in this study are available on request from the corresponding author.
